# Unshared binding sites for *Bacillus thuringiensis* Cry3Aa and Cry3Ca proteins in the weevil *Cylas puncticollis* (Brentidae)

**DOI:** 10.1016/j.toxicon.2016.09.014

**Published:** 2016-11

**Authors:** Patricia Hernández-Martínez, Natalia Mara Vera-Velasco, Baltasar Escriche

**Affiliations:** a Departamento de Genética, Facultad de CC. Biológicas, Universitat de València, Dr Moliner 50, 46100, Burjassot, Spain; b Estructura de Recerca Interdisciplinar en Biotecnologia i Biomedicina (ERI BIOTECMED), Universitat de València, Dr Moliner 50, 46100, Burjassot, Spain

**Keywords:** African sweetpotato weevil, Binding sites, Insect resistance management, Insecticidal proteins, Insect control

## Abstract

*Bacillus thuringiensis* Cry3Aa and Cry3Ca proteins have been reported to be toxic against the African sweetpotato pest *Cylas puncticollis*. In the present work, the binding sites of these proteins in *C. puncticollis* brush border vesicles suggest the occurrence of different binding sites, but only one of them is shared. Our results suggest that pest resistance mediated by alteration of the shared Cry-receptor binding site might not render both Cry proteins ineffective.

## Highlights

• N-terminal sequence of Cry3C activated by trypsin or chymotrypsin was identified at 159 and 153 positions, respectively.
• Cry3Aa and Cry3Ca proteins bound specifically to *C. puncticollis* BBMV.
• Cry3Aa and Cry3Ca proteins do not completely compete for the same binding sites.

## Graphical abstract

**Figure:**
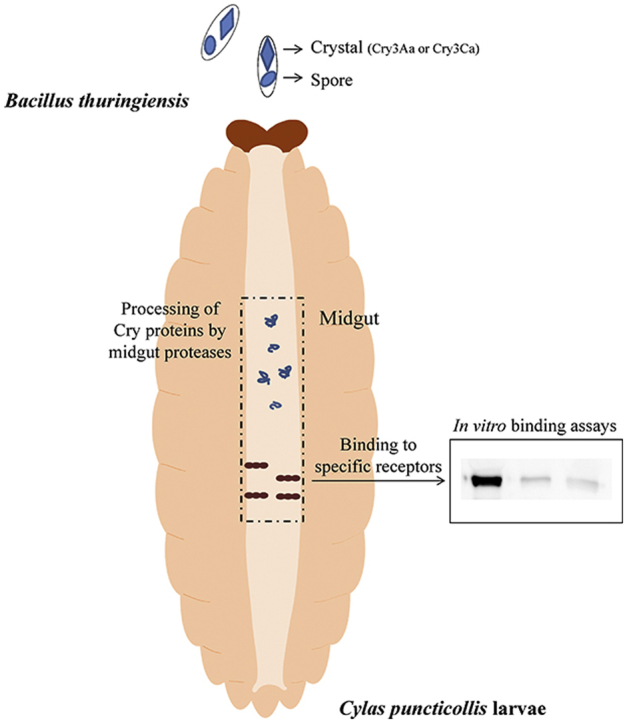

*Cylas puncticollis* (Boheman) (Coleoptera: Brentidae) is one of the major pests of sweetpotato (*Ipomoea batatas* (L.) Lam.) in Eastern Africa (Smit, 1997). The sweetpotato weevil larvae make tunnels in roots and stems causing extensive damage (Stathers et al., 2005) which can cause yield losses of up to 60–100% depending on the severity of the infestation (Chalfant et al., 1990). Chemical control is not effective enough due to the cryptic habit of the larvae (Reddy et al., 2012). Hence, the use of transgenic plants which express *Bacillus thuringiensis* proteins (Bt crops) can be an useful alternative to control *C. puncticollis* insect pest as they have been shown to effectively control stem borers, ear feeders and rootworms (Loseva et al., 2002; Walters et al., 2008). Nowadays, genetically modified sweetpotato plants expressing Cry3Aa, Cry3Ca or Cry7Aa proteins, which have been reported to be active against *C. puncticollis* (Ekobu et al., 2010), have been developed in order to control different sweetpotato weevils of the genus *Cylas* (Morán et al., 1998, Rukarwa et al., 2013a, Rukarwa et al., 2013b).

The mode of action of Cry proteins from *B. thuringiensis* has been extensively studied, especially for lepidopteran-active Cry proteins (Bravo et al., 2007, Vachon et al., 2012; Adang et al., 2014), whereas much less is known for coleopteran-active Cry proteins (Hernández-Martínez et al., 2014, Ochoa-Campuzano et al., 2012, Rausell et al., 2004, Rausell et al., 2007, Slaney et al., 1992). The proposed model starts after the ingestion of the crystals by susceptible insect larvae, followed by crystal solubilization and protease activation in the midgut environment. Finally, the toxic fragment binds, as a key step, to specific receptors on the brush border membrane of the midgut epithelium columnar cells and that leads to toxin insertion into the membrane producing lytic pores which causes cell lysis and insect death (Federici et al., 2010).

The study of the Cry binding site model can help to maintain the long-term efficacy of Bt-crops, since binding site alteration has been described as the basis of cross-resistance when different Cry proteins share the same binding site (Ferré and Van Rie, 2002; Ferré et al., 2008). Recently, the existence of common binding sites for three *B. thuringiensis* proteins, Cry3Ca, Cry3Bb, and Cry7Aa proteins to *C. puncticollis* brush border membrane vesicles (BBMV) has been proposed (Hernández-Martínez et al., 2014). Thus, from a resistant management standpoint, combinations of these three proteins do not seem to be suitable for development of Bt sweetpotato plants. However, there is no information available about Cry3Aa protein binding sites in *C. puncticollis*. For this reason, the aim of the present study was to assess whether Cry3Aa and Cry3Ca proteins share binding sites in this pest to predict possible cross-resistance patterns for these Cry proteins which have been already introduced separately into sweetpotato plants.

Cry3Aa and Cry3Ca proteins were obtained from the *B. thuringiensis* strains BGSC-4AA1 (provided by ARS Culture Collection) and BTS02109P (provided by Bayer CropScience, Gent, Belgium), respectively. Cry3 protein solubilization and activation, either with bovine pancreas trypsin (type I) (Sigma-Aldrich) or bovine pancreas α-chymotrypsin was performed as described by Hernández-Martínez et al. (2014). Processing of Cry3Aa protoxin with either trypsin or chymotrypsin renders a single main polypeptide with a mass of about 55 kDa (Fig. S1). Similar results were described previously, though a second fragment of about 49 kDa was also described to occur together with the 55 kDa fragment when Cry3Aa protoxin was processed *in vitro* with chymotrypsin (Carroll et al., 1997, Martínez-Ramírez and Real, 1996). These differences could be attributed to differences in the experimental conditions used.

Processing of the Cry3Ca protein with either trypsin or chymotrypsin renders a fragment with a mass of about 53 kDa (Rausell et al., 2004) (Fig. S1). Additionally, processing of Cry3Ca protein by either *C. puncticollis* gut fluid or BBMV also rendered a fragment with a mass of about 53 kDa (Martínez-Solís et al., 2011).

Cry3 proteins (73 kDa) are considered as truncated versions of the lepidopteran-active proteins (130 kDa) (Park et al., 2009). However, to be active the Cry3 protoxins must be processed at the N-terminal part of the protein (Carroll et al., 1989, Rukmini et al., 2000). In general, it has been proposed that serine proteases such as trypsin-like or chymotrypsin-like proteases are involved in the processing of *B. thuringiensis* Cry protoxins (Carroll et al., 1989, Carroll et al., 1997, Mohan and Gujar, 2003, Oppert et al., 1996). In the present study, the N-terminal sequence of either trypsin or chymotrypsin-activated Cry3Ca proteins was determined as described by Hernández-Martínez et al. (2014). Briefly, protein bands were cut out from the membrane and sent for N-terminal amino acid sequencing by the Edman method at the Alphalyse A/S, Odense, Denmark, using an ABI Procise 494 sequencer. The N-terminal sequence of the trypsin-activated fragments was SQGRI, corresponding to the position 159, whereas the N-terminal sequence of the chymotrypsin-activated fragment was TLRDG at the position 153. The N-terminal sequences of the trypsin- and chymotrypsin-activated Cry3Aa proteins was described by Carroll et al. (1997) and correspond to the aminoacid positions 159 (sequence NPHSQ) and 162 (sequence SQGRI), respectively.

Previous studies (Slaney et al., 1992, Rausell et al., 2004) have shown that some Cry3 proteins are able to bind to BBMV from some coleopteran insect pest including *C. puncticollis* (Hernández-Martínez et al., 2014). Interestingly, some reports have shown that only the chymotrypsin-activated Cry3Aa, and not the trypsin-activated, was able to bind specifically to BBMV from *L. decemlineata* (Martínez-Ramírez and Real, 1996). In contrast, Rausell et al. (2004) did not observe differences in the binding ability of either trypsin- or chymotrypsin Cry3Aa protein to BBMV from the same insect species. To clarify the active binding fragment for Cry3Aa and Cry3Ca proteins in *C. puncticollis*, competition assays were carried out with either trypsin- or chymotrypsin-activated proteins. BBMV were prepared from whole last-instar *C. puncticollis* larvae based on the differential magnesium precipitation method (Wolfersberger et al., 1987) as modified by Escriche et al. (1995). Trypsin- and chymotrypsin-activated Cry3 proteins were biotinylated with a protein biotinylation kit (GE HealthCare) according to the manufacturer's instructions. The working conditions for the binding experiments were set up in preliminary experiments. Competition experiments were performed incubating 5 μg of BBMV with 18 nM of biotinylated trypsin or chymotrypsin-activated Cry3 proteins in binding buffer (phosphate-buffered saline, pH 7.4, 0.1% BSA) in the absence or the presence of an excess of unlabeled Cry proteins. Incubations were carried out for 1 h at 25 °C in a final volume of 100 μl. Moreover, control binding assays conducted without BBMV showed practical absence of protein precipitation (Fig. S2).

At least three replicates were performed to each competition assay. Binding was detected as previously described by Hernández-Martínez et al. (2014) using streptavidin-conjugated horseradish peroxidase (1:2000).

Homologous competition assays showed that either trypsin or chymotrypsin-activated Cry3Aa and Cry3Ca proteins bound specifically to the *C. puncticollis* BBMV since they exhibited competition with an excess of their respective unlabeled Cry protein (Fig 1). In order to test the role of proteolytic processing by commercial enzymes on binding ability, labeled Cry3Aa and Cry3Ca trypsin or chymotrypsin-activated proteins were competed with unlabeled chymotrypsin or trypsin-activated Cry3Aa or Cry3Ca proteins, respectively. In all cases, the results showed a similar reduction on the binding of the biotinylated Cry3 proteins suggesting that independently of the protease treatment the Cry3 protein binds to the same receptor (Fig 1).

Thus, the differences in the N-terminal sequence described by other authors to either trypsin- or chymotrypsin-activated Cry3Aa (Carroll et al., 1997) or by ourselves to either trypsin- or chymotrypsin-activated Cry3Ca are not essential for the binding ability at least in *C. puncticollis.*

Heterologous competition binding assays were performed with the chymotrypsin-activated Cry3 proteins. The results showed that the majority of the labeled Cry3Aa was competed off by a 400-fold excess of Cry3Ca (Fig. 2A). Unlabeled Cry3Aa was not able to completely displace labeled Cry3Ca when a 400-fold excess was used in the assay (Fig 2B). These results suggest that Cry3Aa and Cry3Ca proteins may have two different binding sites and only one of them is shared among them. Thus, an alteration of the shared binding site in *C. puncticollis* might not confer resistance to both proteins, since both Cry3 proteins have an unshared binding site. The occurrence of shared binding sites on *C. puncticollis* BBMV for two Cry3 proteins (Cry3Bb and Cry3Ca) was previously described (Hernández-Martínez et al., 2014). Moreover, this common binding site is also shared with the Cry7Aa protein. Shared binding sites for three Cry3 proteins were also reported on Colorado potato beetle BBMV (Rausell et al., 2004). Nevertheless, this is the first study that demonstrated the occurrence of shared and unshared binding sites between Cry3Aa and Cry3Ca proteins.

In summary, based on the results of binding site interactions, the development of cross-resistance between Cry3Aa and Cry3Ca proteins due to a single binding site modification appears to be unlikely in *C. puncticollis*, since both proteins have unshared binding sites. Thus, from a resistant management standpoint, combinations of Cry3Aa and Cry3Ca can be suitable for development of Bt sweetpotato plants.

## Figures and Tables

**Fig. S1:**
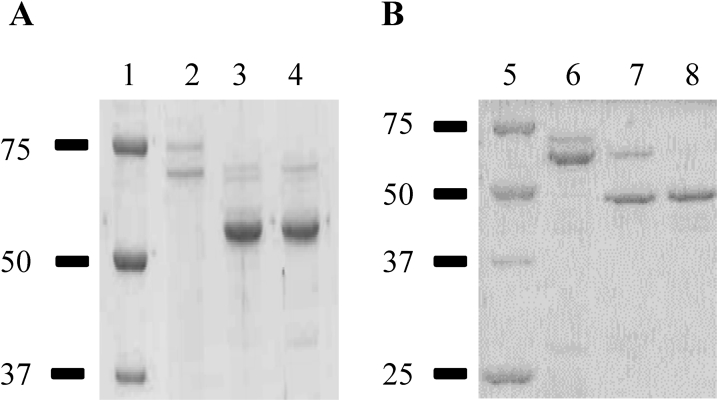
SDS-PAGE (12%) analysis of the protease-resistant fragments obtained after trypsin or chymotrypsin processing of the solubilized Cry3Aa (**A**) or Cry3Ca (**B**) protoxins. Lanes 1 and 5, molecular mass marker (size labeled in kD) a); lanes 2 and 6, Cry3Aa and Cry3Ca protoxins, respectively; lanes 3 and 7, activation with trypsin; lanes 4 and 8, activation with chymotrypsin.

**Fig. S2:**
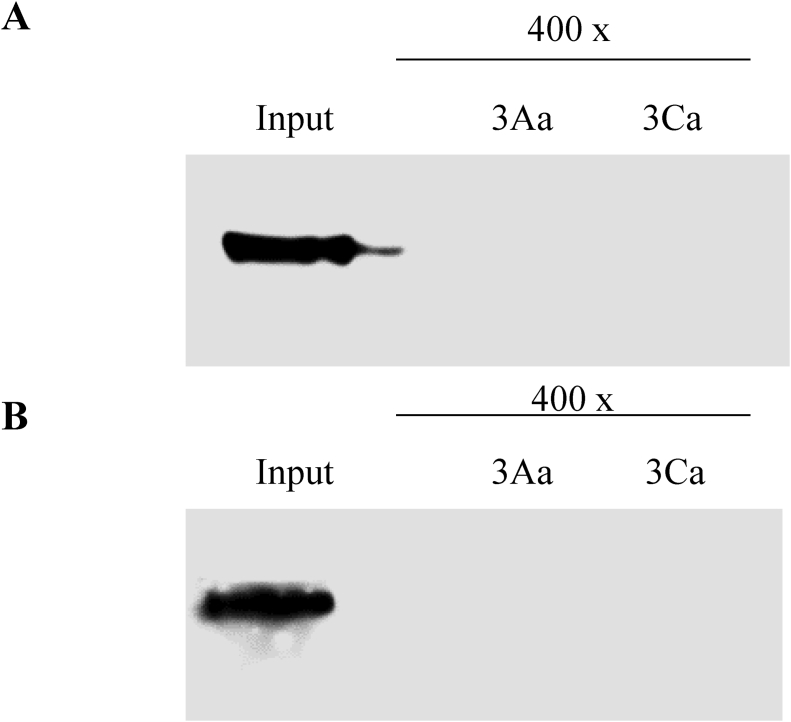
Control experiments of the protein precipitation on binding assays. Biotinylated chymotrypsin-activated Cry3Aa (**A**) or Cry3Ca (**B**) proteins were incubated in absence of *C. puncticollis* BBMV and in the presence (400x) of unlabeled chymotrypsin-activated proteins. Input indicates biotinylated Cry3 proteins without BBMV.

**Fig. 1:**
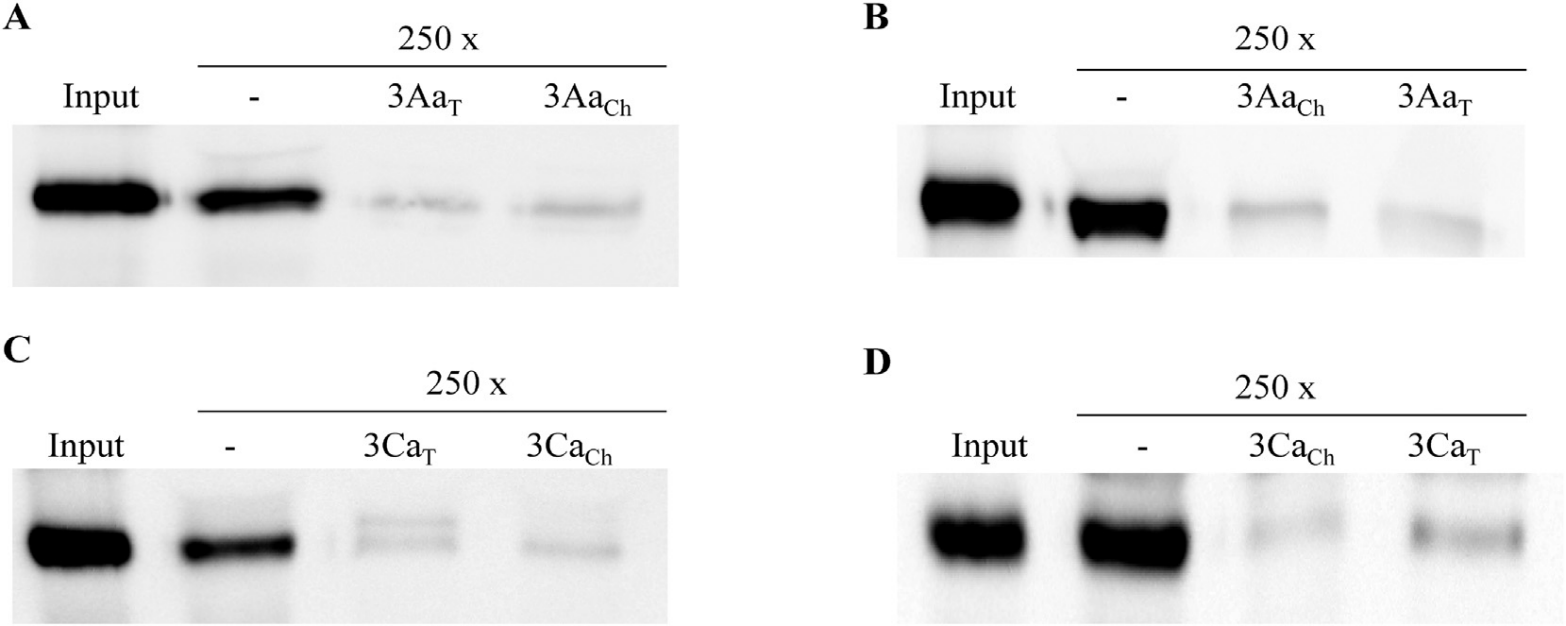
Homologous competition binding assays on *C. puncticollis* BBMV. Biotinylated trypsin or chymotrypsin-activated Cry3 proteins were incubated in absence (−) or presence (250x) of unlabeled trypsin or chymotrypsin-activated proteins. **A**, biotinylated trypsin-activated Cry3Aa (3Aa_T_); **B**, biotinylated chymotrypsin-activated Cry3Aa (3Aa_Ch_); **C** biotinylated trypsin-activated Cry3Ca (3Ca_T_); **D**, biotinylated chymotrypsin-activated Cry3Ca (3Ca_Ch_). Input indicates biotinylated Cry3 proteins.

**Fig. 2:**
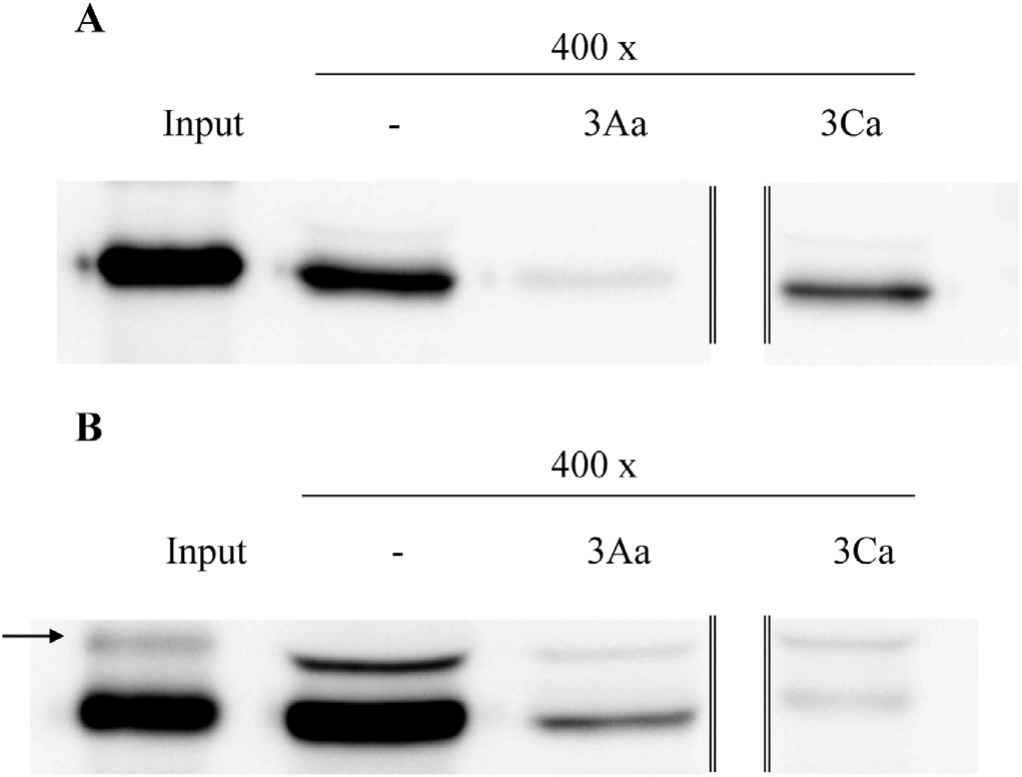
Heterologous competition binding assays on *C. puncticollis* BBMV. Biotinylated chymotrypsin-activated Cry3 proteins were incubated in absence (−) or presence (400x) of unlabeled chymotrypsin-activated proteins. **A**, biotinylated Cry3Aa; **B**, biotinylated Cry3Ca. All lanes came from a single experiment, but the vertical lines indicate that these lanes were not consecutive. The arrow indicated the presence of not fully processed Cry3 labeled protein. Input indicates biotinylated Cry3 proteins.
